# Intrinsically Conductive Films of Ag_2_Te Nanocrystals for Broadband Infrared Photodetection beyond 2000 nm

**DOI:** 10.1021/acs.nanolett.6c03525

**Published:** 2026-09-17

**Authors:** Yuanfei Huo, Yulia V. Kuznetsova, Anastasiia V. Sokolova, Chenxi Guo, Aleksandr A. Sergeev, Kam Sing Wong, Kseniia A. Sergeeva, Andrey L. Rogach

**Affiliations:** † Department of Materials Science and Engineering and Centre for Functional Photonics (CFP), 53025 City University of Hong Kong, 83 Tat Chee Avenue, Kowloon, Hong Kong SAR 999077, People’s Republic of China; ‡ Institute of Solid State Chemistry of the Ural Branch of the Russian Academy of Sciences, 91 Pervomaiskaya Str., Ekaterinburg 620990, Russia; § Department of Physics and William Mong Institute of Nano Science and Technology, The Hong Kong University of Science and Technology, Clear Water Bay, Hong Kong SAR 999077, People’s Republic of China; ∥ School of Science and Engineering and Guangdong Basic Research Center of Excellence for Aggregate Science, Chinese University of Hong Kong, Shenzhen, Guangdong 518172, People’s Republic of China

**Keywords:** silver telluride, nanocrystals, aprotic solvent, hot-injection synthesis, short-wavelength infrared photodetection

## Abstract

We report a rapid, up-scalable synthesis in an aprotic solvent to obtain Ag_2_Te nanocrystals that are intrinsically capped with a short, conductive thiol ligand, making them suitable for optoelectronic applications. The simultaneous use of labile and stable tellurium precursors controlled the reaction kinetics and enabled growth of nonbranched, highly crystalline Ag_2_Te nanocrystals, with well pronounced excitonic peaks and an absorption edge extending beyond 2000 nm. We also realized synthesis of either n-type or p-type Ag_2_Te nanocrystals by simply tuning their stoichiometry. Using Ag_2_Te nanocrystal inks, we fabricated infrared photodetectors in a phototransistor configuration, with a high responsivity above 2 A/W and a specific detectivity under broadband illumination reaching 10^11^ Jones at 160 K, and rise and fall times of 50 and 80 μs, fast enough to resolve kilohertz-scale optical modulation. Our study encourages development of heavy-metal-free nanocrystals for infrared optoelectronic devices operating at temperatures accessible to commercial Peltier coolers.

## Graphical abstract

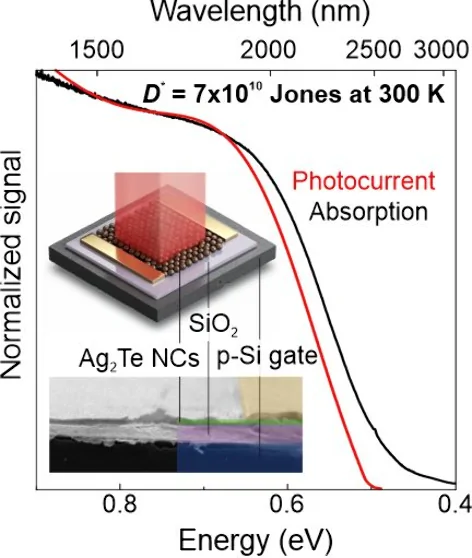

Colloidal silver telluride (Ag_2_Te) nanocrystals (NCs) have recently emerged as eco-friendly, narrow-bandgap, solution-processable semiconductors for infrared (IR) optoelectronics.^1,2^ Their bulk bandgap is predicted to be within the range of 0.06–0.67 eV,^1,3–5^ enabling size-tunable absorption across the near-IR and short-wave IR range (SWIR). Especially their low toxicity relative to lead- and mercury-based counterparts makes Ag_2_Te NCs highly attractive for real-world applications. Recently, Ag_2_Te NC-based photodiodes reached detectivities of 10^10^–10^12^ Jones in SWIR (1400–1800 nm),^5–7^ and up to 10^9^ Jones when the absorption cutoff was extended toward mid-wave IR range.^8,9^ While those photodetectors have already approached figures-of-merits of state-of-the-art Pb- and Hg-chalcogenide SWIR detectors,^1,10^ several challenges remain. In particular, the reported devices showed elevated dark current at room temperature and rather slow response, both of which remain open challenges for SWIR imaging.^10^ Another fundamental issue is the poor redox stability of Ag_2_Te NCs,^11^ since high mobility of silver ions makes their films labile to bias-driven redox changes, which distorts electrical response and, in extreme cases, results in nucleation of metallic silver clusters leading to the shortage of devices.

Similar to many other colloidal NCs, Ag_2_Te NCs are commonly synthesized with long-chain insulating ligands, which must subsequently be exchanged for short-chain ligands to improve the conductivity of their close-packed films.^7^ Such postsynthetic treatment usually requires several consecutive steps, complicating device fabrication, and may result in incomplete surface passivation, leaving NCs prone to oxidation and limiting their long-term stability. A variation of the cation-to-anion ratio at the NC surface calls for a different choice of suitable ligands, thus requiring more sophisticated strategies for the ligand exchange.^12^ Eliminating this step would be important for subsequent fabrication of reliable and up-scalable fabrication Ag_2_Te NC-based optoelectronic devices.

In this work, we report a rapid, up-scalable synthesis of Ag_2_Te NCs in an aprotic medium, yielding NCs which are capped with short-chain thiol ligands already at the synthesis stage, which ensures an intrinsic conductivity of the NC films without any need for postpreparative surface treatment. Optimizing the synthesis parameters provided Ag_2_Te NC inks with a well-defined excitonic feature at around 2000 nm. Using those inks as the active material in field-effect transistors (FETs), we demonstrate high responsivity above 2 A/W and a specific detectivity of 10^11^ Jones under broadband SWIR illumination at 160 K, and rise and fall times of 50 and 80 μs,respectively, fast enough to resolve kilohertz-scale optical modulation.

To produce Ag_2_Te NCs, we followed the continuous growth of the NCs in aprotic solvent approach, which was previously developed^13^ and refined^14^ for HgTe NCs. Following this synthetic route, we used tripropylamine (TPA) to stabilize H_2_Te gas, which was slowly and continuously supplied with the TPA-HTe adduct in a flask containing dimethylformamide (DMF), silver nitrate, and 2-furanmethanethiol (FMT) as a ligand (Figure 1a). Although the obtained Ag_2_Te NCs showed good colloidal stability, they were branched, with a broad size distribution and poor crystallinity, as evidenced from transmission electron microscopy (TEM) images in Figure 1b. Their excitonic feature could only be pushed to 1500 nm before it broadened and eventually disappeared due to Ostwald ripening (Figure 1c). This is because the TPA-HTe adduct is highly labile and depleted immediately upon addition, causing the monomer concentration to drop right after the nucleation burst, so that the NC growth stage could not be maintained. Rapid supply of the whole TPA-HTe volume in one go further confirmed that the entire precursor nucleated at once, producing NCs with a featureless absorption edge (dashed curve in Figure 1c).

**Figure 1:**
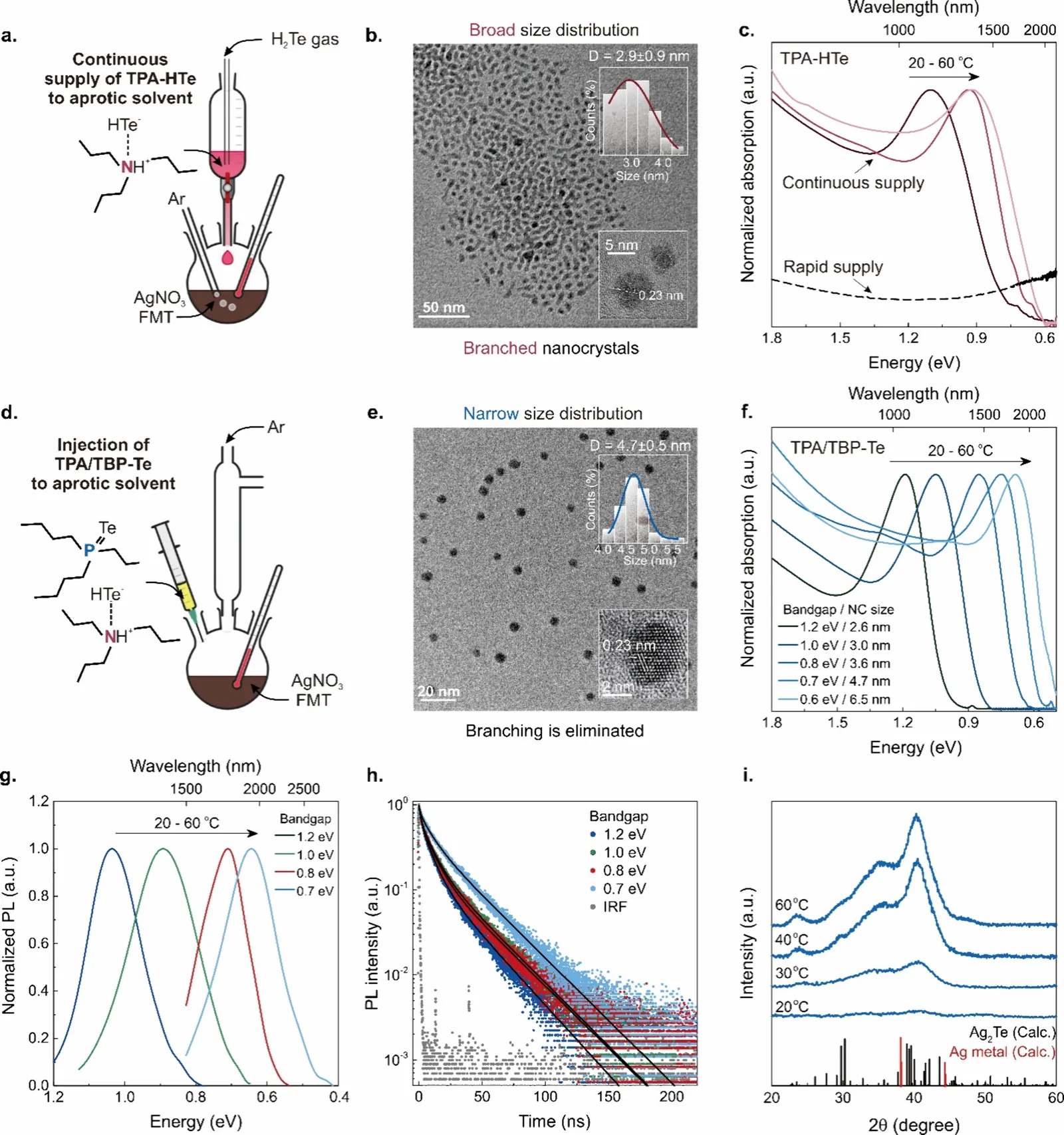
**Synthesis and optical properties of Ag**_**2**_ **Te NCs in an aprotic solvent, using different tellurium precursors. a.** A scheme of slow and continuous supply of TPA-HTe adduct to a flask containing AgNO_3_ and FMT ligand. **b.** TEM image of Ag_2_Te NCs obtained using the continuous supply of TPA-HTe precursor in DMF. The insets show a high-resolution TEM image of a single NC and the size distribution of the produced NCs. **c.** Absorption spectra of Ag_2_Te NCs obtained using slow and continuous supply of TPA-HTe adduct (solid curves) or rapid supply of TPA-HTe adduct (dashed curve). **d.** A scheme of aprotic hot-injection approach: a TPA/TBP-Te precursor is injected into a flask containing AgNO_3_ and FMT ligand. **e.** TEM image of Ag_2_Te NCs obtained using the TPA/TBP-Te precursor. A high-resolution TEM image of a single NC and size distribution of the produced NCs are shown in the insets. **f.** Absorption spectra of Ag_2_Te NCs obtained using the TPA/TBP-Te precursor (corresponding values of the bandgap and NC sizes are presented in the legend). **g.** Red-shift of PL spectra with decrease of Ag_2_Te NC bandgap. Synthesis temperatures are also provided for reference. **h.** PL decay curves (dots) of Ag_2_Te NCs with different bandgaps presented in panel **g**. The data was fitted with triexponential function (black solid lines). IRF stands for instrument resolution function. **i.** Evolution of monoclinic crystal phase of Ag_2_Te in XRD patterns with increasing synthesis temperature. Black and red bars represent reference XRD patterns calculated for bulk Ag_2_Te and metal Ag, respectively.

To provide a reliable supply of the monomers for controllable growth of Ag_2_Te NCs, we combined labile TPA with a stable tributylphosphine (TBP) as a buffering organic base (Figure 1d). Since TBP has a limited solubility in polar solvents, toluene was added to DMF (in a v:v ratio of 1:10) to homogenize the mixture. This ensured a complete capture of the H_2_Te gas by the TPA/TBP mixture in DMF, but because of a negligible volume of toluene used, this did not affect the colloidal stability of the produced NCs. Moreover, we used a single rapid injection of the TPA/TBP-Te precursor rather than a continuous supply. TPA-HTe and TBP-Te precursors dissociate at different rates, which allowed us to separate the nucleation and growth stages in time. Upon injection, the weakly bound TPA-HTe adduct provided monomer supersaturation and nucleation of Ag_2_Te NCs. At the same time, the more robust TBP-Te complex ensured a steady monomer supply for the controlled growth of the existing NCs instead of triggering new nucleation. The NCs obtained by injection of TPA/TBP-Te to aprotic solvent showed a more negative ζ-potential (down to −40 mV) and consequently longer colloidal stability than those synthesized by the continuous supply of TPA-HTe (Figure S1).

Among several reaction parameters that were proven to be critical (Figure S1), the molar ratio of the two organic bases was the most important one. The use of a 1:1 molar TPA/TBP mixture yielded uniform, nonbranched spherical Ag_2_Te NCs with well-defined lattice fringes (Figures 1e and S2) and sharp excitonic peaks spanning the whole spectral range of 1000 to 2000 nm and slightly beyond (Figure 1f). The optical bandgap values extracted from the absorption spectra (Figure S3) correlated with the NC sizes obtained from TEM images; the bandgap decreased from 1.2 to 0.7 eV when the NC size increased from 2.5 to 4.7 nm. Fitting the size-dependent bandgap values of Ag_2_Te NCs with the generalized expression^15^ yielded bulk E_g_ = 0.4 eV and a Bohr exciton diameter of d_Bohr_ = 20 nm (Figure S4a). This bulk bandgap agrees well with previously reported estimates,^1,3–5^ while the Bohr diameter is roughly twice the commonly cited values for silver chalcogenides.^1,5^ We also noticed that the Urbach energy, which is a measure of band-tail disorder, remained within a narrow range (18–26 meV) across the same NC bandgap series (Figure S4b), which is comparable to values reported for other low-toxicity colloidal materials.^16^

Silver chalcogenides obtained in polar solvents typically exhibit weak photoluminescence (PL) with a large Stokes shift, attributed to the presence of structural defects.^12,17,18^ In our case, in stark contrast, the PL band of Ag_2_Te NCs was narrow and symmetric (Figure 1g), with a quality factor (defined as the ratio of the fwhm of the PL band to its peak wavelength) of 25 ± 5% (Table S1). Based on PL decays (Figures 1h and S5, Table S2), the average PL lifetime increased from 17.5 to 25.4 ns (for PL maxima at 1200 and 1750 nm, respectively). This is consistent with the improved crystallinity of larger NCs, as confirmed by XRD data (those grown at higher temperatures, Figures 1h and S6). On the other hand, this trend does not exclude a contribution from defect states, since the PL intensity followed a power law with a slope of 0.75 ± 0.03 across the full incident-fluence range for all the samples (Figure S5). We acknowledge that the underlying physics of such behavior is more complex than our present data may imply, and we reserve this for future studies.

Altering the Ag:Te ratio can significantly affect electronic structure and intrinsic doping of Ag_2_Te NCs. Revealing those effects is a crucial point for fabricating efficient NC-based photodetectors. Thus, for further studies we prepared Ag_2_Te NCs with Ag:Te ratios of 4:1 and 2:1 and a bandgap of 0.6 eV (corresponding to the absorption cutoff beyond 2000 nm, and therefore sensitive over the SWIR region). The experimental atomic composition of the samples was verified by survey X-ray photoelectron spectroscopy (XPS). The core-level peaks of Ag, Te, S, C, and O were clearly resolved in the survey spectra (Figures 2a and S7). Notably, Ag:Te ratios determined by XPS were 4.1 ± 0.3 and 2.3 ± 0.1 (Table S3), indicating a reasonable fluctuation within the synthetic load. We will further denote those samples as Ag-rich (4:1) and st-Ag_2_Te (2:1; “st” stays for stoichiometric ones).

**Figure 2:**
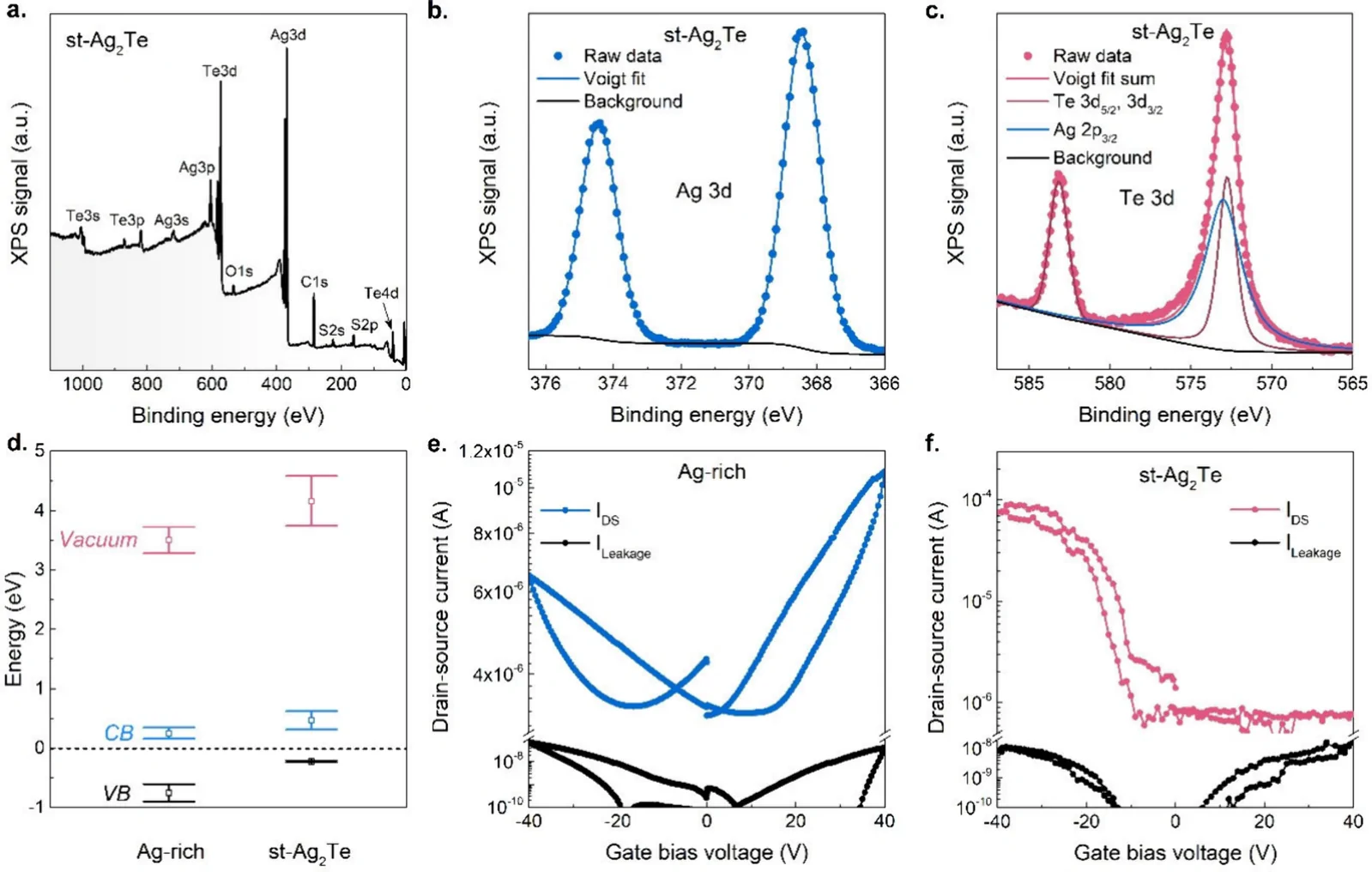
**Electronic and electrical properties of Ag**_**2**_ **Te NCs obtained by injection of TPA/TBP-Te precursor to aprotic solvent. a.** XPS survey spectrum of st-Ag_2_Te NCs. **b.** Ag 3d and **c.** Te 3d core-level high-resolution XPS spectra of st-Ag_2_Te NCs and their fits with Voigt functions. **d.** Positions of the valence band (VB), conduction band (CB) and vacuum level with respect to Fermi level (set to zero, dashed line) determined by UPS for Ag-rich and st-Ag_2_Te NCs. **e.** and **f.** Transfer curves under a constant drain-source bias of −20 V for FETs fabricated from Ag-rich (**e.**) and st-Ag_2_Te (**f.**) NCs, measured at 260 K.

Importantly, the high-resolution XPS spectrum of Ag 3d showed broad peaks that lack any metallic Ag^0^ component, confirming structural stability of all types of Ag_2_Te NCs under study (Figures 2b and Figure S8a), respectively. The shape of the Te 3d spectra, by contrast, strongly depended on NC stoichiometry: the peaks were well pronounced in st-Ag_2_Te samples but nearly vanished in Ag-rich ones (Figure 2c and Figure S8b, respectively). The absence of any Te–O or Ag–O features further confirmed that the produced material was resistant to oxidation, thanks to robust surface passivation. In addition, XPS analysis detected no amine- or phosphine-derived species (Figure S7) in the studied samples, which may be due to the strong affinity of Ag for the thiol groups; this makes FMT the dominantly present ligand at the NC surface, while TPA and TBP species could be readily removed during purification.

By performing ultraviolet photoemission spectroscopy (UPS), we revealed that Ag-rich NCs were predominantly *n*-type, owing to their cation-rich surface and the presence of FMT ligand at the surface (Figure 2d). Along with passivating under-coordinated Ag sites, the thiolate from FMT introduces a molecular dipole at the surface, which shifts the vacuum level and bends the bands near the surface, tuning the electron affinity and promoting the *n*-type character of the NCs.^19^ On the other hand, for st-Ag_2_Te NCs the Fermi level was found to lie closer to the valence band (Figure 2d). This tunability was reflected in the transfer curves of bottom-gated field effect transistors (FETs) measured at 260 K (Figure 2e and 2f). In this configuration, Ag-rich (*n*-type) NC films showed ambipolar-like behavior with pronounced hysteresis on both the positive and negative gate bias arms. In turn, st-Ag_2_Te NC films (*p*-type) exhibited transfer curves with a weaker hysteresis. Hole mobilities reached 0.5 and ∼10^–4^ cm^2^ V^–1^ s^–1^ for *p*-type and *n*-type NCs, respectively (Figure S9). We notice that the electron mobility for Ag-rich NCs was of the same order of magnitude (∼10^–4^ cm^2^ V^–1^ s^–1^ at 260 K) as for holes, which, despite the modest value, indicated more efficient charge balance compared with st-Ag_2_Te NCs. Based on this observation, we selected this sample to be used as an active layer for a SWIR photodetector.

Using the same FET geometry, we characterized the IR photoresponse of a device consisting of preprinted Au electrodes, a *p*-doped Si gate, and a SiO_2_ dielectric (Figure 3a), with Ag-rich NCs as an active layer, whose absorption cutoff extended beyond 2000 nm (Figure 3b). Since FMT is a short, conductive ligand, these Ag_2_Te NCs did not require any pre- or postdeposition ligand exchange. This allowed us to easily convert the purified NCs into highly concentrated (>50 mg/mL) inks, which were spin-coated as four successive layers, yielding a uniform 100 nm-thick film (Figure 3a). Notably, spectrally resolved photocurrent reproduced the shape of the absorption spectrum of the NC film with a slight blue-shift (∼50 nm) of the absorption cutoff (Figure 3b).

**Figure 3:**
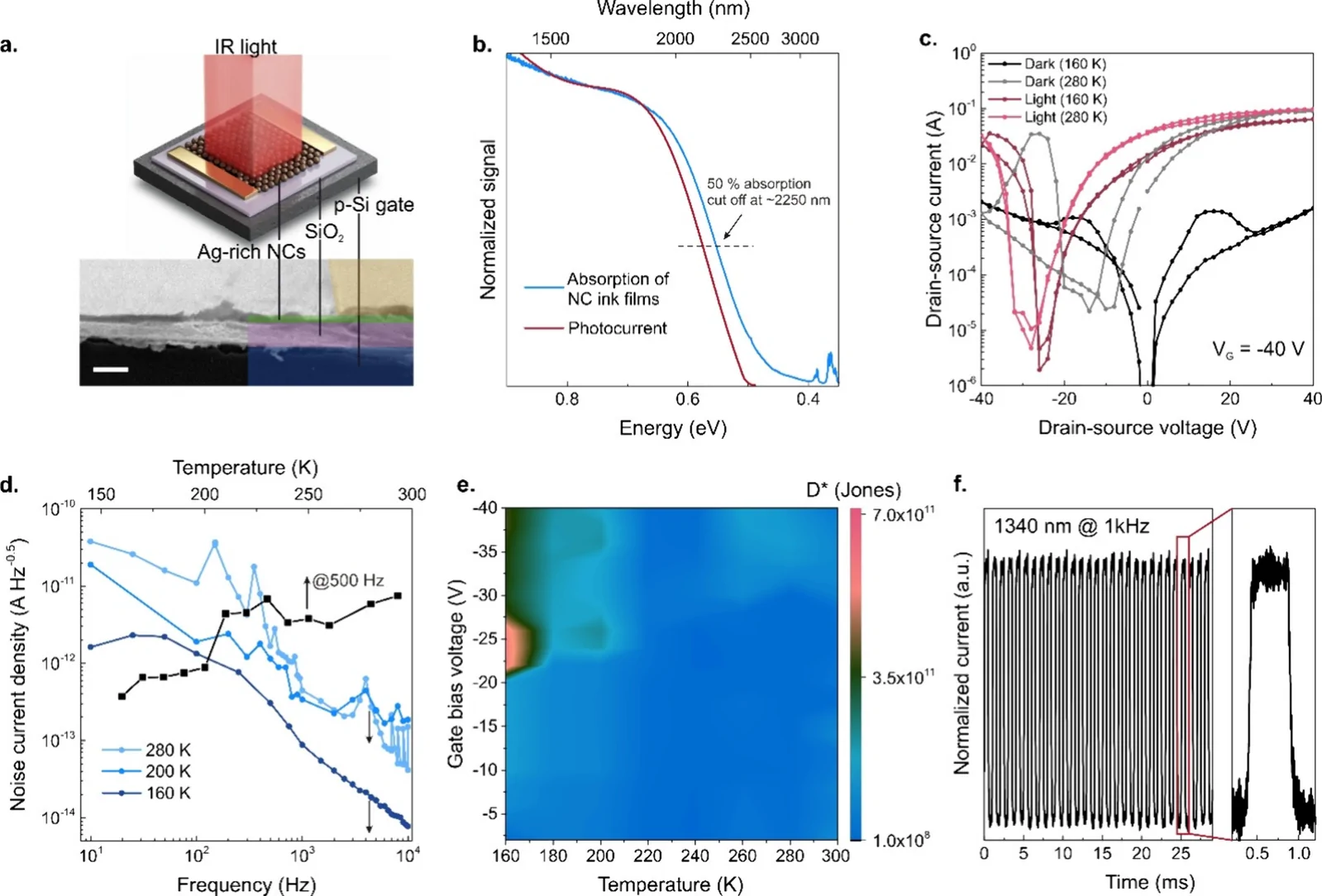
**Broadband SWIR photodetection using Ag-rich Ag**_**2**_ **Te NCs. a.** Schematics of the FET with Ag-rich NCs forming the drain-source channel; the cross-sectional SEM image of this device visualizes the *p*-Si gate (blue false color), the SiO_2_ dielectric layer (pink color), the NC active layer (green color), and the gold contact (yellowish color). The scale bar is 300 nm. **b.** Absorption spectra of the film made of NC inks (50 mg/mL) and FET photocurrent under V_DS_ = 20 V and V_G_ = −20 V. **c.** Output I_DS_–V_DS_ characteristics of the FET under dark and illuminated (“light”) conditions, at 160 and 280 K. A calibrated 2796 K blackbody was used as the light source, with a 1000 nm long-pass filter removing its visible component. **d.** Noise current density (*S*_*n*_) spectra at 160, 200, and 280 K (blue curves) and the temperature dependence of *S*_*n*_ at a fixed frequency of 500 Hz (black curve). **e.** Specific detectivity map of the FET as a function of gate bias voltage and operating temperature. **f.** Transient photocurrent of the FET under illumination by a 1340 nm laser modulated with an optical chopper at 1 kHz. The zoom-in on the right shows a single dark/light cycle in detail.

Figure 3c shows the output characteristics (I_DS_–V_DS_) of the FET photodetector at V_G_ = −40 V, recorded at 160 and 280 K in the dark, and under illumination by a 2796 K calibrated blackbody filtered through a 1000 nm long pass. The dark current density estimated at 160 K under V_DS_ = 40 V and V_G_ = −40 V was 0.15 A cm^–2^, which is expected for a FET operating in the saturation regime. In the dark, the output curves at both temperatures displayed electrochemical signatures, which we attribute to reduction of mobile Ag^+^ ions at the electron-injecting contact (Ag^*+*^ + e^*–*^ → Ag^0^), whose release was facilitated by the cation-rich NC surface.^11^ At 160 K the current minimum lay near V_DS_ ≈ 0 V, whereas at 280 K it shifted to about −10 V, as the migration of thermally activated Ag^+^ ions enhanced the Faradaic contribution to the current and enabled redistribution of the ions under the gate-induced field. Under illumination, the electrochemical features vanished, likely due to reoxidation of Ag^0^ by photogenerated holes. However, the current minimum shifted further toward negative V_DS_, while its thermal dependence was preserved. This suggests that, even once the Faradaic process is suppressed, the gate field continues to redistribute Ag^+^ ions and, together with photogating by the trapped holes, shifts the channel potential. Meanwhile, the large photocurrent at 160 K resulted in a broadband responsivity as high as 2.7 A/W, depending on the applied gate bias voltage (Figure S10a). This value fell by an order of magnitude (∼0.1 A/W) near room temperature but, to our knowledge, still represents the highest responsivity reported so far for Ag_2_Te NC-based phototransistors (Figure S11).

The noise current density spectra were dominated by 1/f noise across the measured frequency range and increased with temperature between 160 and 280 K (Figure 3d). At 1 kHz, V_DS_ = 40 V, and V_G_ = −20 V, the specific detectivity D* to blackbody illumination reached 3 × 10^11^ Jones at 160 K (Figure 3e), and it remained above 7 × 10^10^ Jones even at noncryogenic temperatures (260–300 K) in the FET saturation regime (Figure S10b).

Finally, the temporal response was measured under square-wave modulation of a 1340 nm laser. The photocurrent shown in Figure 3f was stable and uniform from cycle to cycle, confirming reproducible switching of the photodetector. Within a single cycle, however, the response was not perfectly square-shaped. We defined the rise time as the interval over which the signal increased from 10% to 90% of its steady-state value and the fall time as the interval over which it dropped back from 90% to 10%. These values were 50 and 80 μs, respectively, which is fast enough to resolve kilohertz-scale optical modulation and shorter than the response times reported so far for Ag_2_Te NC-based phototransistors.^20,21^

In summary, we report a simple and efficient injection-to-a-protic-solvent route that was developed to obtain monodisperse, size-tunable Ag_2_Te NCs. One key advantage of our approach is that these NCs were intrinsically capped with a short, conductive thiol ligand and thus required no further surface treatment to be suitable for device integration. These NCs were found to be resistant to oxidation and colloidally stable owing to charge stabilization between the ligands and the aprotic solvent. By tuning the stoichiometry of Ag_2_Te NCs toward an excess of cation, the Fermi level could be shifted toward the conduction band, promoting n-type doping of these NCs. Close-packed films of Ag-rich NCs exhibited high carrier mobility, together with a pronounced, reversible electrochemical response in the dark. The FET infrared photodetector showed a broad spectral response with a cutoff beyond 2000 nm and reached broadband specific detectivities above 10^11^ Jones and 10^10^ Jones at 160 and 300 K, respectively. To the best of our knowledge, this represents the most sensitive Ag_2_Te NC-based phototransistor operating beyond 2000 nm reported to date. Future work will focus on improving the device architecture and exploring a photovoltaic-type configuration with high-quality interfaces for more efficient carrier extraction. Overall, the results reported here encourage the development of heavy-metal-free NC optoelectronics operating at temperatures accessible to commercial Peltier coolers.

## Supplementary Material



## Notes

Methods; synthesis refinement; size-dependent optical bandgap and Urbach energy; photoluminescence properties; atomic composition; charge carrier mobility; thin film morphology; temperature-dependent responsivity and detectivity (PDF)
