# Supplementary material for: Intrinsically Conductive Films of Ag2Te Nanocrystals for Broadband Infrared Photodetection beyond 2000 nm
Source: Nano Lett. 2026 Sep 17;26(38):13071–7. doi: 10.1021/acs.nanolett.6c03525 (PMC13632580; doi:10.1021/acs.nanolett.6c03525)
Supplement: Supplementary file 1 [file nl6c03525_si_001.pdf]

Supporting Information

Intrinsically Conductive Films of Ag_2Te Nanocrystals for Broadband Infrared Photodetection beyond 2000 nm

Yuanfei Huo[1], Yulia V. Kuznetsova[1,2], Anastasiia V. Sokolova[1], Chenxi Guo[1], Aleksandr A. Sergeev[3], Kam Sing Wong[3,4], Kseniia A. Sergeeva[1,*] and Andrey L. Rogach[1,*]

[1]Department of Materials Science and Engineering, and Centre for Functional Photonics (CFP), City University of Hong Kong, 83 Tat Chee Avenue, Kowloon, Hong Kong SAR 999077, People's Republic of China

[2]Institute of Solid State Chemistry of the Ural Branch of the Russian Academy of Sciences, 91 Pervomaiskaya Str., Ekaterinburg 620990, Russia

[3]Department of Physics and William Mong Institute of Nano Science and Technology, The Hong Kong University of Science and Technology, Clear Water Bay, Hong Kong SAR, 999077, People's Republic of China

[4]School of Science and Engineering and Guangdong Basic Research Center of Excellence for Aggregate Science, Chinese University of Hong Kong, Shenzhen, Guangdong, 518172, People's Republic of China

E-mail: ksergeeva2@cityu.edu.hk and andrey.rogach@cityu.edu.hk

Table of Contents

1. Methods 2
2. Synthesis refinement for the injection to aprotic solvent approach towards Ag_2Te NCs 6
4. Photoluminescence properties of Ag_2Te NCs 9
5. Refinement of XRD patterns 11
6. Atomic composition of Ag_2Te NCs 12
7. Photodetector characterization 14

1. Methods

Chemicals. Silver nitrate ($AgNO_3$, Sigma Aldrich, 99.0 %), 2-furanmethanthiol (FMT, Sigma Aldruch, 98%), 65 % phosphoric acid, N,N-dimethylformamide (DMF, AQA, 99.8 %), tripropylamine (TPA, Sigma Aldrich, 98 %), tributylphosphine (TBP, Aladdin, 95 %), tributylamine (TBA, ThermoFisher, 98 %), 2-methylimidazole (2-MIZ, Macklin, 98%), ethyl acetate (EA, AQA, 99.5 %), 1-hexanol (HEX, Aladdin, 99 %), acetonitrile (ACN, ACI Labscan, 99.9 %), dimethyl sulfoxide (DMSO, DUKSAN, 99.0 %), 1-dodecanethiol (DDT, Sigma Aldrich, 98 %), tetrachloroethylene (TCE, ACI Labscan, 99.8 %), isopropyl alcohol (IPA, AQA, 99.0 %), argon gas (Ar, 99.999 %, Linde).

Preparation of TPA-HTe and TPA/TBP-Te precursors. TPA, TBP, or the mixture of these two substances were dissolved in DMF and transferred into a pressure-equalising funnel. H_2Te gas was generated in an electrochemical cell at a solid tellurium electrode (cathode), while O_2 was generated at a platinum wire (anode), with 65% phosphoric acid acting as the electrolyte. Each new tellurium electrode and/or stabilizer was calibrated to estimate the yield η of the electrochemical cell according to the formula:

$$\eta = \frac{2F \times n}{I \times t}, \quad (1)$$

where $F = 96485$ s A mol^{-1} is the Faraday constant; n is the amount of H_2Te (mol); I is the current (A); and t is the time (s).

The typical efficiency of our electrochemical cell was 60% at 300 mA. H_2Te gas was carried out of the electrochemical cell by a regulated flow of Ar gas. The colour of the solution in the funnel changed from pale yellow to dark purple during gas saturation, due to the reaction of the organic base (TPA and TBP) with H_2Te gas, which resulted in the formation of an organo-hydrogen telluride TPA-HTe adduct and more stable TBP-Te compound. No any grey precipitate was formed during the H_2Te saturation, meaning that the telluride precursor was effectively and completely captured by the stabilizer. The gas outlet from the funnel was vented into a measuring cylinder containing a 1M NaOH solution to absorb any excess H_2Te gas that would not fully react. Had this occurred, the colour of the NaOH solution would have turned to pink, then deep purple, and finally grey upon prolonged contact with air. Freshly prepared tellurium precursor was used straightaway for the continuous growth procedure of Ag_2Te NCs, or transferred to a nitrogen-filled glovebox for storage.

Synthesis of Ag_2Te NCs using the continuous growth approach. In a three-neck flask, 5 mmol of $AgNO_3$ together with 10 mmol of FMT deprotonated by 2-MIZ was dissolved in 30 ml of DMF. In a pressure-equalizing funnel mounted on the three-neck flask and attached to the electrochemical cell, 50 ml of DMF and 2 ml of TPA were mixed. Both vessels were purged with dry argon gas for 1 h. H_2Te gas was saturated into the funnel for 25 min. Then, 10 ml of the TPA-HTe adduct was added quickly dropwise into a cold (-13 °C) cation precursor. The rest of the tellurium precursor was added dropwise in a slow, controlled manner, with a simultaneous ramp of the temperature.

Synthesis of Ag_2Te NCs by injection in an aprotic solvent. In a three-neck flask, 5 mmol of $AgNO_3$ was dissolved in 30 ml of DMF. The flask was evacuated and refilled with argon three times to remove moisture. Following argon purging, the system was heated to the target temperature (between 20 and 80 °C). Then, 10 mmol of FMT deprotonated by 2-MIZ were injected at 10 °C below the target temperature. The tellurium precursor (TPA-Te, TBP-Te or TPA/TBP-Te) which was prepared in advance was injected into the flask upon reaching the target temperature. The reaction was maintained for 10 min, after which the flask was cooled in an ice bath.

Purification of Ag_2Te NCs. 5 ml of crude Ag_2Te NC solution in DMF was placed in a centrifuge tube. An equal amount of hexane was added, and EA was added dropwise until hexane and DMF started to mix. The

solution was vortexed for 1 min and centrifuged at 5000 rpm for 10 min. The supernatant was discarded, and the pellet obtained as a precipitate by centrifugation was gently dried under argon flow. Then the required amount of DMF was added to obtain either highly concentrated inks (>50 mg/ml) or diluted Ag_2Te NC solutions for optical measurements.

Synthetic yield estimation. The synthetic reaction yield was determined gravimetrically with respect to tellurium. After the synthesis, the NCs were precipitated with ACN, separated by centrifugation, and washed 3 times to remove unreacted precursors and excess ligands, and dried to constant weight. The yield was calculated according to the formula:

$$Y = \frac{m_{NCs}}{n_{Te} \times M_{Ag_2Te}} \times 100\ \%, \tag{2}$$

where m_{NCs} is the mass of the dried purified product, n_{Te} is the total amount of tellurium introduced with the TPA-HTe and TBP-Te precursors, and M_{Ag_2Te} = 343.3 g mol^{-1}. Since the ligand shell cannot be removed completely by washing, the values obtained in this way represent an upper estimation of the true yield.

Transmission electron microscopy (TEM) measurements were conducted on a JEOL JEM-2100F microscope at an accelerating voltage of 200 keV. The samples were purified and diluted more than 500 times before applying them on the carbon-coated copper grids.

X-ray and ultraviolet photoelectron spectroscopy (XPS and UPS, respectively). Ag_2Te NC ink was drop-cast on a pre-cleaned gold substrate and kept under primary vacuum overnight prior to the experiment. XPS and UPS spectra were measured on an Escalab Xi+ (Thermo Scientific) spectrometer with AlKα source (λ=8.3386 Å). The data collection area was 900 µm. The pass energy was set to 100 and 20 eV for the survey and high-resolution scans, respectively. Backgrounds were subtracted using the Shirley or Tougaard functions, and the X-ray absorption cross-section of each component (Ag 3d, Te 3d or 4d, S 2p, C 1s, O 1s) was taken into account when calculating the atomic ratios from high-resolution spectra. Note, that for the samples with Ag:Te ratio of 4:1 (Ag-rich) the signal from Te 3d electrons nearly vanished or could not be reliably distinguishable from the Ag $2p_{3/2}$ peak. Therefore, we used Te 4d peak to estimate the composition.

The workfunction value, W_f, was estimated using the formula $W_f = KE_{Cut\text{-}off} - PolBias$, where $KE_{Cut\text{-}off}$ is the secondary electron cut-off energy, and *PolBias* is the polarization bias (5 V). The position of the valence band was estimated using the formula $E_v - E_F = h\upsilon - KE_{vb} - W_{fa}$, where E_v is the valence band position, E_F is the Fermi energy, $h\upsilon$ is the energy of the He I source (21.2 eV), KE_{vb} is the kinetic energy, and W_{fa} is the spectrometer workfunction.

X-ray diffraction (XRD). XRD patterns were recorded in parallel beam mode using a Rigaku SmartLab 9kW diffractometer with $CuK\alpha_{1,2}$ radiation source, which was equipped with a sample stage allowing for height correction. The samples were drop-cast onto a low-background silicon substrate and dried under primary vacuum.

UV-Vis-IR absorption, steady state and time-resolved photoluminescence (PL) spectroscopy. UV-Vis-IR absorption spectra were collected using a Shimadzu 3600i Plus spectrometer in an IR-grade quartz cuvette with an optical path of 1 cm. PL spectra were recorded on an FLS920P spectrometer system (Edinburgh Instruments), which was equipped with a liquid-nitrogen-cooled InSb photodiode and an 880 nm continuous wave solid-state laser as the excitation source. Time-resolved PL decays were collected using a purpose-built optical setup. Output light from a tunable Ti:sapphire femtosecond oscillator (Coherent Mira900; <130 fs pulse width, 76 MHz repetition rate, 800 nm central wavelength) was pulse-

picked down to a 2 MHz repetition rate, and the pulse fluence was kept below 1 nJ/cm^2 to avoid any multi-excitonic processes. The PL signal from the sample was collimated and directed towards the input of a fiber-coupled IR photon detection module based on a cooled InGaAs/InP single-photon avalanche photodiode. An 860 nm long-pass filter was used to block any scattered pump light. To ensure a single-photon counting mode, the photon hit-rate was kept below 2% of the pulse repetition rate.

Fabrication of photodetectors and electrical measurements. For the bottom-gate FET fabrication, commercially available substrates (n-doped Si wafers with a 300-nm-thick silica gate layer and pre-printed interdigitated gold electrodes with 13 channels, each 5 mm long and 10 μm wide, from Suzhou Crystal Silicon Electronic & Technology Co. Ltd., China) were used. The substrates were cleaned sequentially in deionized water/soap, acetone, and IPA for 10 min, each step in an ultrasonic bath, and then placed in an ozone cleaner for 30 min. A 20 μL aliquot of the Ag_2Te NC ink was spread onto the substrate and spin-coated at 2500 rpm for 90 s in air. The process was repeated four times to reach the desired film thickness of ~100 nm.

FET devices were inserted into an Optistat DN2 cryostat (Oxford instruments). The sample chamber was evacuated and backfilled with dry argon gas, repeating this process three times to ensure the chamber was free from oxygen and moisture. A Mercury iTC (Oxford Instruments Company) unit was used to control the cryostat temperature in the range between 300 and 80 K. Electrical measurements were conducted using two Keithley 2440 source meters controlled by a LabVIEW program to apply bias voltages to the gate-source and drain-source channels and record the current. Voltage scans were performed in the forward and reverse mode in the range from +40 to -40V, each cycle starting and ending at 0 V. For the photoresponse measurements, a calibrated blackbody at 2796 K (quartz-tungsten lamp, Thorlabs) was used as the illumination source. The part of the blackbody spectrum at wavelengths shorter than 1000 nm was cut off with a long-pass filter, while the 50 % point of the Ag_2Te NC optical absorption edge at 2250 nm served as the long-wavelength limit. The incident power of the light source was calculated using the equation:

$$P = \pi A_{\mathrm{det}}^2 sin\left(\frac{\theta}{2}\right) cos\varphi \int_{1.0\mu m}^{\lambda_{cut-off}} \frac{2hc^2}{\lambda^5} \frac{1}{\exp\left(\frac{hc}{\lambda kT}\right)-1} d\lambda, \quad (3)$$

where A_{det} is the area of the photodetector, θ is the field of view, ϕ is the incident angle (typically assumed to be 0°), h is the Planck constant, c is the speed of light, k is the Boltzmann constant, and T is the blackbody temperature. The total power, calculated according to equation (3) taking into account transmission of two sapphire windows and the long-pass filter, was 35 mW.

Current noise measurements were performed on an SRS570 low-noise current pre-amplifier and a SR830 Lock-in amplifier (Stanford Research Systems). Devices were biased from a switchable bank of batteries, and a LabVIEW interface program was used to collect the data, with each point averaged over 5 readings.

The charge carrier mobility (in units of cm^2 V^{-1} s^{-1}) in the linear regime was calculated using the equation:

$$\mu = \frac{L}{W \cdot V_{ds}} \times \frac{1}{C_i}\left(\frac{\partial I_{ds}}{\partial V_g}\right), \quad (4)$$

where W is the width of the electrodes (13 electrode fingers with 5 mm overlap width each), L is the channel length (10 μm), and C_i is the capacitance of the 300 nm SiO_2 gate dielectric layer, measured using a high-precision inductance, capacitance, and resistance meter and determined as $3.5 \cdot 10^{-9}$ F cm^{-2}. The hole mobility was extracted from the linear part of the slope of the I_{DS}-V_G curves.

The photoresponsivity R (in units of A W^{-1}) and the specific detectivity D (in units of Jones) of the photodetectors were calculated using the equations:

$$R = \frac{I_{pc}}{P}, \tag{5}$$

$$D^* = \frac{R\sqrt{A_{det}}}{S_n}, \tag{6}$$

where I_{pc} (A) is the photocurrent, and P (W) is the incident power, S_n is the current noise spectral density (in units of A $Hz^{-1/2}$).

Spectrally resolved photocurrent measurements were conducted using a Bentham ISR-300 monochromator equipped with a built-in current pre-amplifier and analog-to-digital converter. The same blackbody (2796 K, Thorlabs) was used as the light source. The output beam was shaped by a combination of CaF_2 lenses. Transient photocurrent measurements were performed under 1340 nm illumination from a CW laser modulated by an optical chopper wheel (1kHz, Thorlabs). A SRS570 low-noise current pre-amplifier (Stanford Research Systems) was used to amplify the signal, which was collected on a Tektronix TBS C1000 digital oscilloscope.

2. Synthesis refinement for the injection to aprotic solvent approach towards Ag_2Te NCs

We first studied how the choice of the organic base would affect the efficiency of the electrochemical cell, which is defined as the fraction of tellurium remained as a stable precursor rather than lost through H_2Te decomposition. Five stabilizers were tested: three organic bases (TPA, TBA, and TBP), their binary mixtures, as well as two additional compounds, FMT and NaMeO. In a typical experiment, H_2Te gas was supplied at 300 mA for 30 min into a flask containing a Cd salt dissolved in DMF, with no ligand added to stabilize the product. The CdTe precipitate was collected, washed three times with ethanol, dried under primary vacuum, and weighed, and the tellurium content was determined from its mass. The cell efficiency was then calculated according to equation (1).

As expected, two amines TPA and TBA provided the highest yields of 60 % (Figure S1a). TBP, in contrast, has a limited solubility in DMF, and therefore performed poorly on its own, with ~40% of the H_2Te captured. Mixing TBP with TPA in toluene/DMF (1:10 v:v) however, restored the caption efficiency to a satisfactory level of 60%. In turn, FMT proved to be inefficient for a different reason: unlike DDT-Te, the FMT-based precursor was highly unstable over time, which limited its practical use. NaMeO also performed poorly, due to its low solubility in DMF.

Note, that because of their lability amine precursors (TPA-HTe and TBA-HTe) can be stored only for a limited period (usually up to one week) even under an inert atmosphere, after which metallic tellurium is found at the bottom of the flask. When freshly prepared, however, they are perfectly suitable for the synthesis. In turn, when tellurium is coordinated not to hydrogen, but to phosphorus in TBP-Te, the precursor is chemically more robust and can be kept under inert conditions for months without noticeable decomposition. Importantly, we observed the same long-term stability for mixed (1:1 molar) TPA/TBP-Te precursor.

Based on these data, we choose TPA and its 1:1 molar mixture with TBP as the organic bases for preparation of tellurium precursors (TPA-HTe or TPA/TBP-Te) The Ag_2Te NCs obtained using TPA-HTe and TPA/TBP-Te precursors showed excellent colloidal stability (zeta-potential between -20 and -40 mV, respectively) and a high synthetic yield of up to 90% (Figure S1b). The choice of tellurium stabilizer also affected the sharpness of the Ag_2Te NC absorption edge, with a TPA/TBP-Te and TBP-Te offering the best results (Figure S1c). We notice that combination of TBA-HTe with FMT produced broader exciton peak (Figure S1d) and was therefore not pursued further. In addition to very limited FMT-Te precursor stability, the use of FMT in the absence of TBP or TPA was insufficient to produce Ag_2Te NCs with a narrow size distribution and distinguishable excitonic feature.

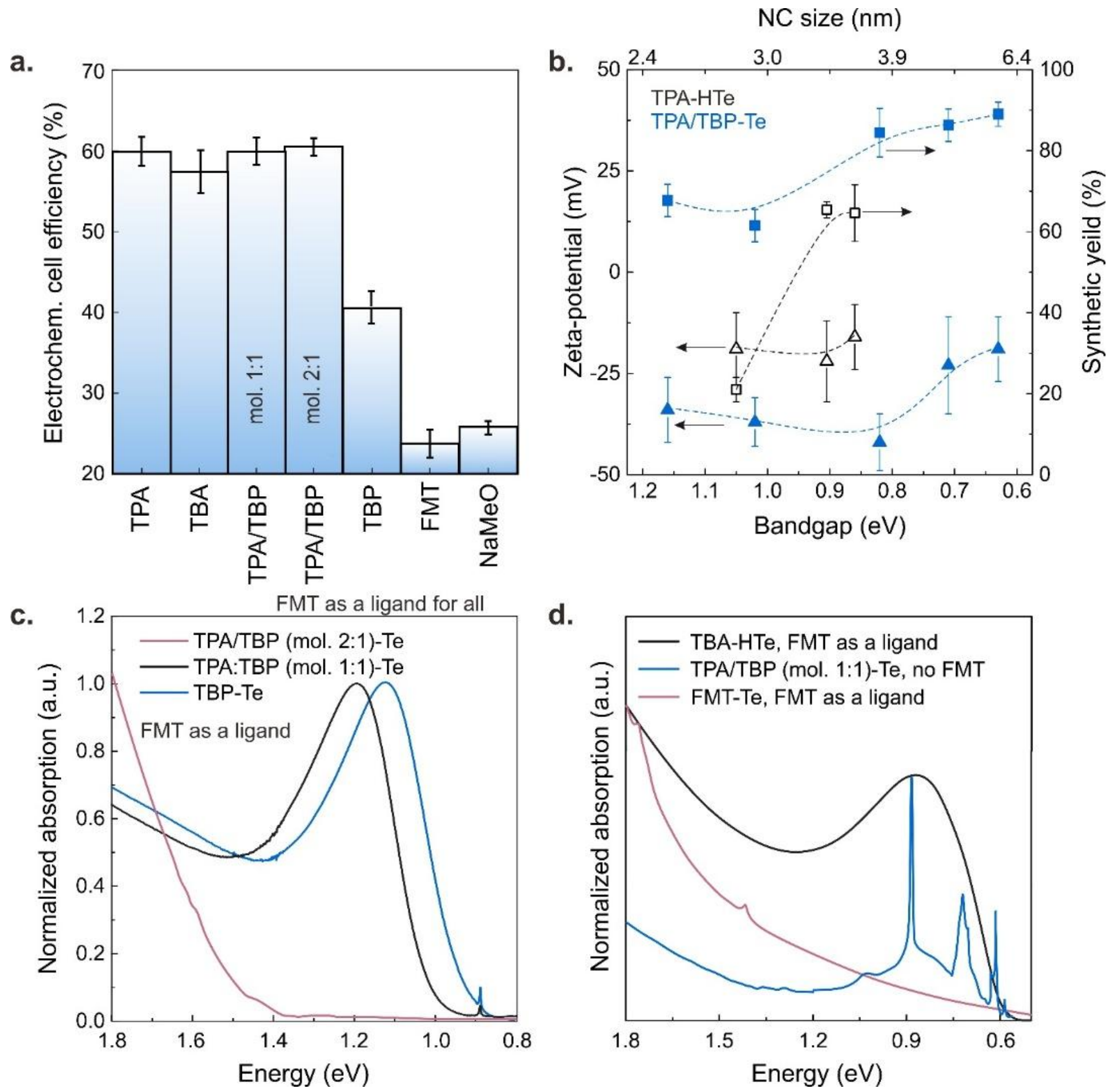

Figure S1. a. The efficiency of the electrochemical cell depending on the chosen organic base. TPA stays for tripropylamine, TBA – for tributylamine, TBP – for tributylphosphine, and FMT – for 2-furanmethanethiol. **b.** Zeta-potentials (triangles) and synthetic yields (squares) for Ag_2Te NCs with different bandgaps; corresponded sizes are provided on the top of X-axis. Dashed B-spline lines are drawn to guide the eye. **c.** and **d.** Absorption spectra of Ag_2Te NCs obtained at 20 °C using different precursor types.

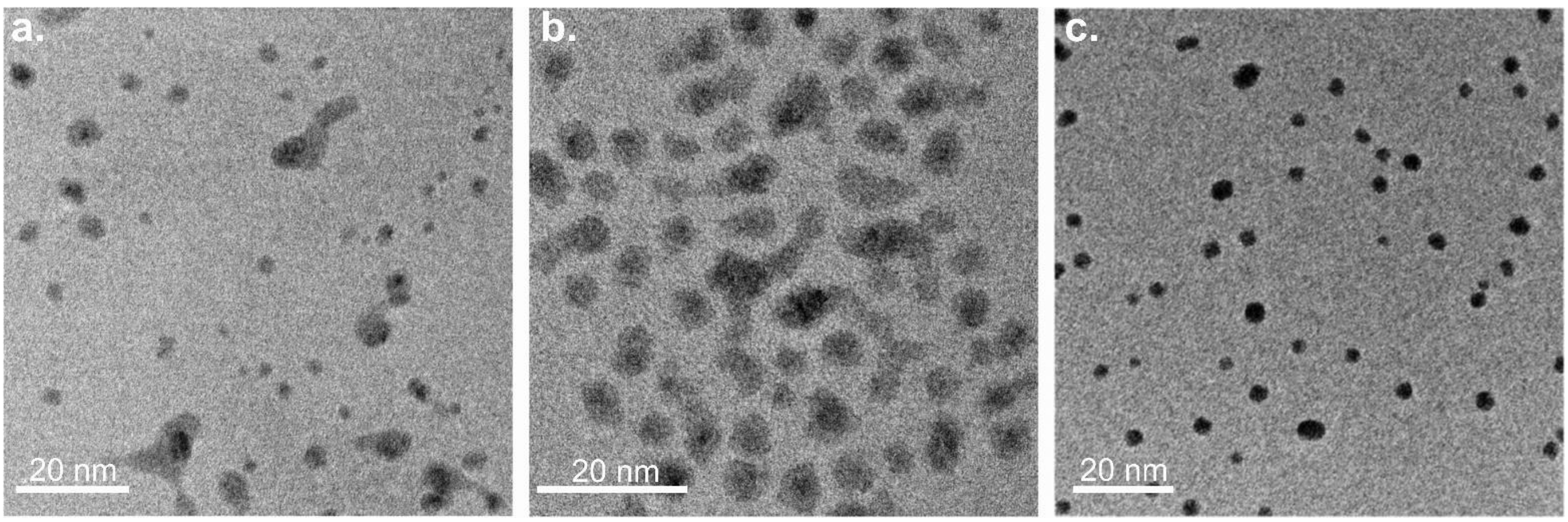

Figure S2. TEM images of Ag_2Te NCs obtained with the TPA/TBP-Te precursor at (a) 20 °C, (b) 40 °C and (c) 60 °C. For the smaller NCs the organic ligand shell is clearly apparent as a low-contrast halo around each particle, which obscures its boundary.

3. Optical bandgaps and Urbach energies of Ag_2Te NCs

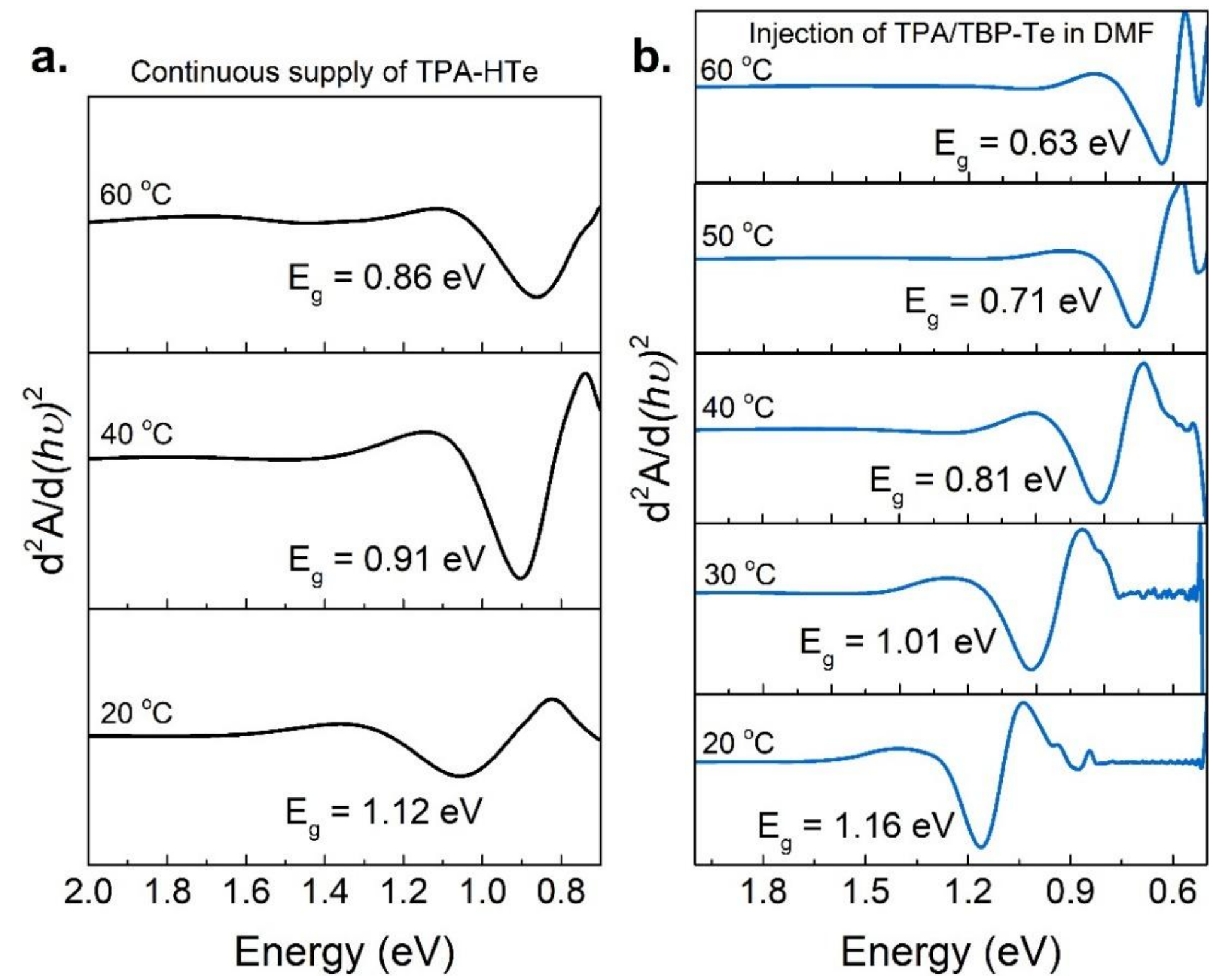

Figure S3. Second derivatives of the absorption spectra shown in Figures 1c and f for Ag_2Te NCs obtained **a.** by continuous supply of TPA-HTe and **b.** injection of TPA/TBP-Te to aprotic solvent. The values of the bandgap E_g were determined from the minima of the second derivatives.

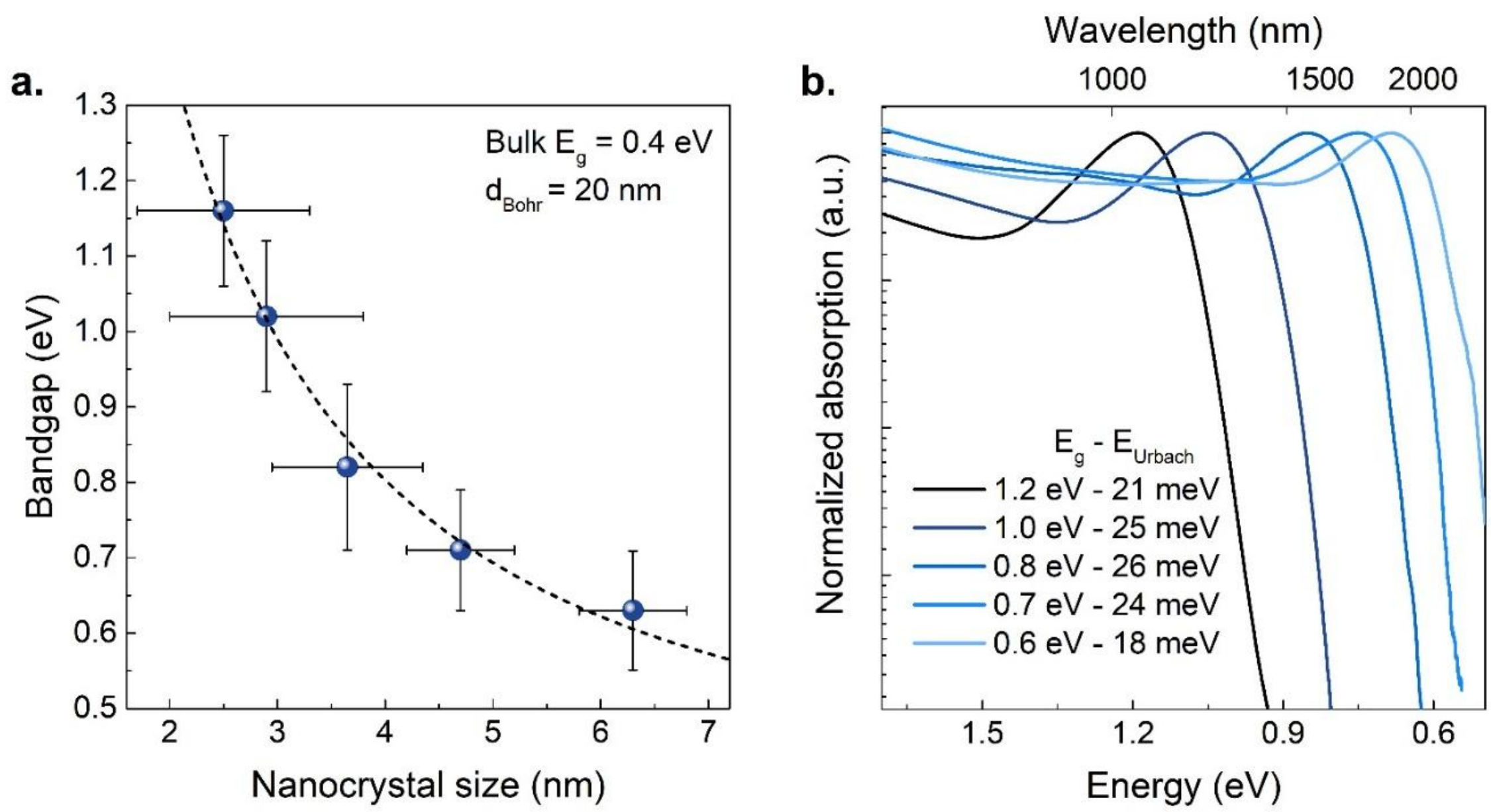

Figure S4. a. Dependence of the bandgap (circles) on Ag_2Te NC size, fitted with the generalized expression from ref.[11] (dashed line). The error bars for the NC size were estimated based on TEM data, while those for the bandgap correspond to the broadening of the first exciton peak. **b.** Urbach energy determined from the absorption spectra shown in Figure 1f. The bandgap values in the legend corresponds to NCs sizes as follows: 2.5 nm (1.2 eV), 3.0 nm (1.0 eV), 3.6 nm (0.8 eV) 4.7 nm (0.7 eV), and 6 nm (0.6 eV).

4. Photoluminescence properties of Ag_2Te NCs

The PL spectra (Figure 1g) were fitted using a Gaussian function, and the fitting parameters are summarized in the Table S1. The PL decay curves for a series of Ag_2Te NCs shown in Figure 1i were fitted using triexponential function. The fitting parameters are listed in the Table S2.

Table S1. Fitting parameters of PL spectra shown in Figure 1g, for Ag_2Te NCs with different bandgaps.

Parameters	NC bandgap in [eV] and corresponding size in [nm]			
	1.2 eV (2.5 nm)	1.0 eV (3.0 nm)	0.8 eV (3.6 nm)	0.7 (4.7 nm)
PL maximum [eV]	1.0	0.9	0.7	0.6
Stokes shift [eV]	0.2	0.1	0.1	0.1
PL linewidth [eV]	0.2	0.2	0.2	0.2
Quality factor (FWHM/PL max) [%]	20	22	28	33

Table S2. Fitting parameters of PL decay curves shown in Figure 1h, for Ag_2Te NCs with different bandgaps.

Components of decay fit	NC bandgap in [eV] and corresponding size in [nm]			
	1.2 eV (2.5 nm)	1.0 eV (3.0 nm)	0.8 eV (3.6 nm)	0.7 (4.7 nm)
A_1	0.3	0.3	0.3	0.2
τ_1 [ns]	1.7	1.2	1.4	1.3
A_2	0.5	0.5	0.5	0.3
τ_2 [ns]	7.9	8.4	8.3	9.0
A_3	0.2	0.2	0.2	0.5
τ_3 [ns]	26.7	29.2	29.7	29.6
**$\langle \tau \rangle$ [ns]**	**17.5**	**21.3**	**20.5**	**25.4**

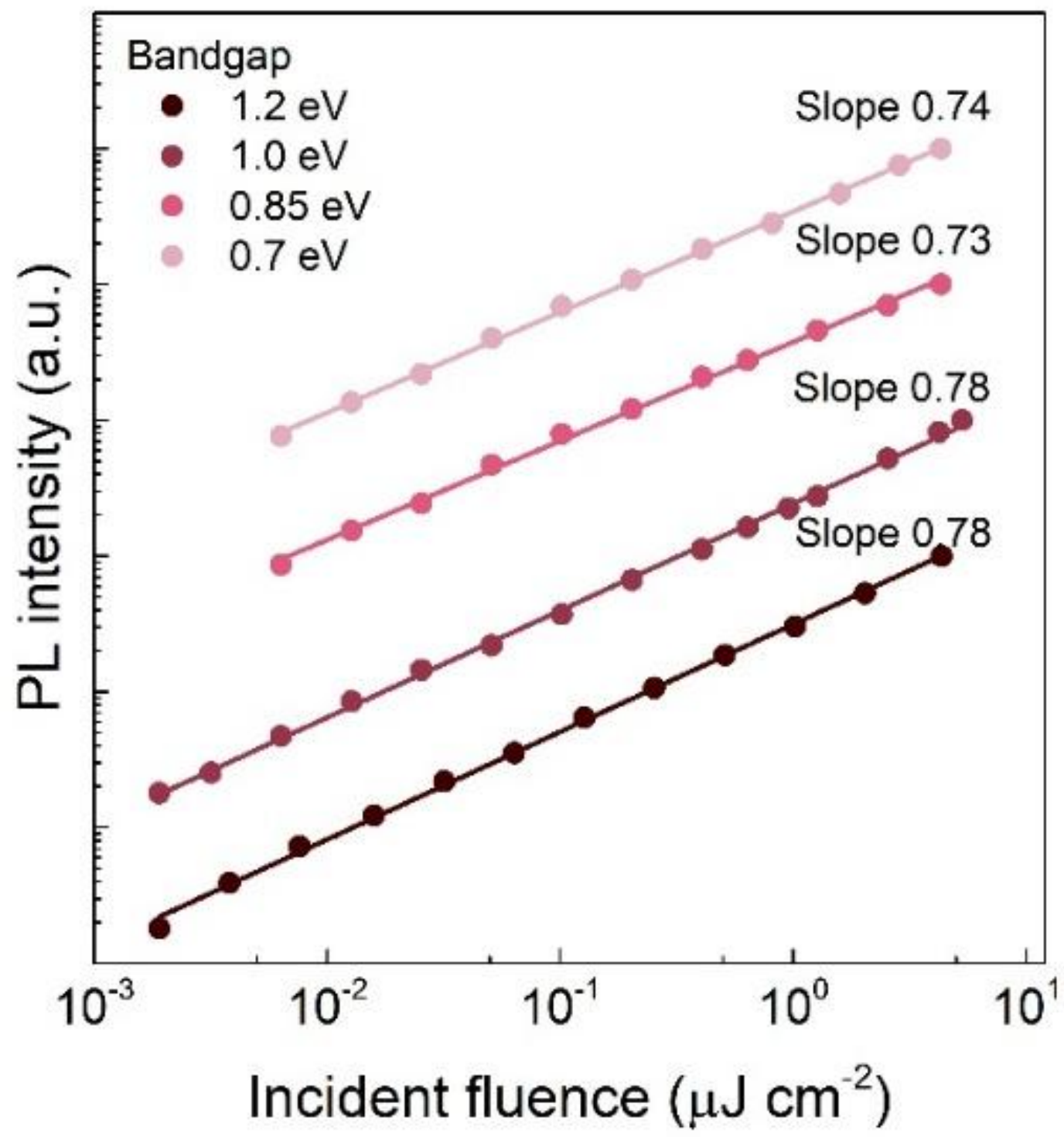

Figure S5. Slopes of PL decays as a function of the incident fluence, for Ag_2Te NC with different bandgaps. Note that the PL decay curves shown in Figure 1h of the main text correspond to maximum incident fluence presented here.

5. Refinement of XRD patterns

The XRD data show that the Ag_2Te NCs adopt monoclinic phase, space group $P2_1/n$, and that the crystallinity improves significantly as the NC size increases. The deviations of the experimental data from the reference peak positions and intensities are attributed to the small crystallite size (<5 nm), which broadens and weakens the reflections, and to the accompanying surface stress, which relaxes the lattice and gives rise to strain.

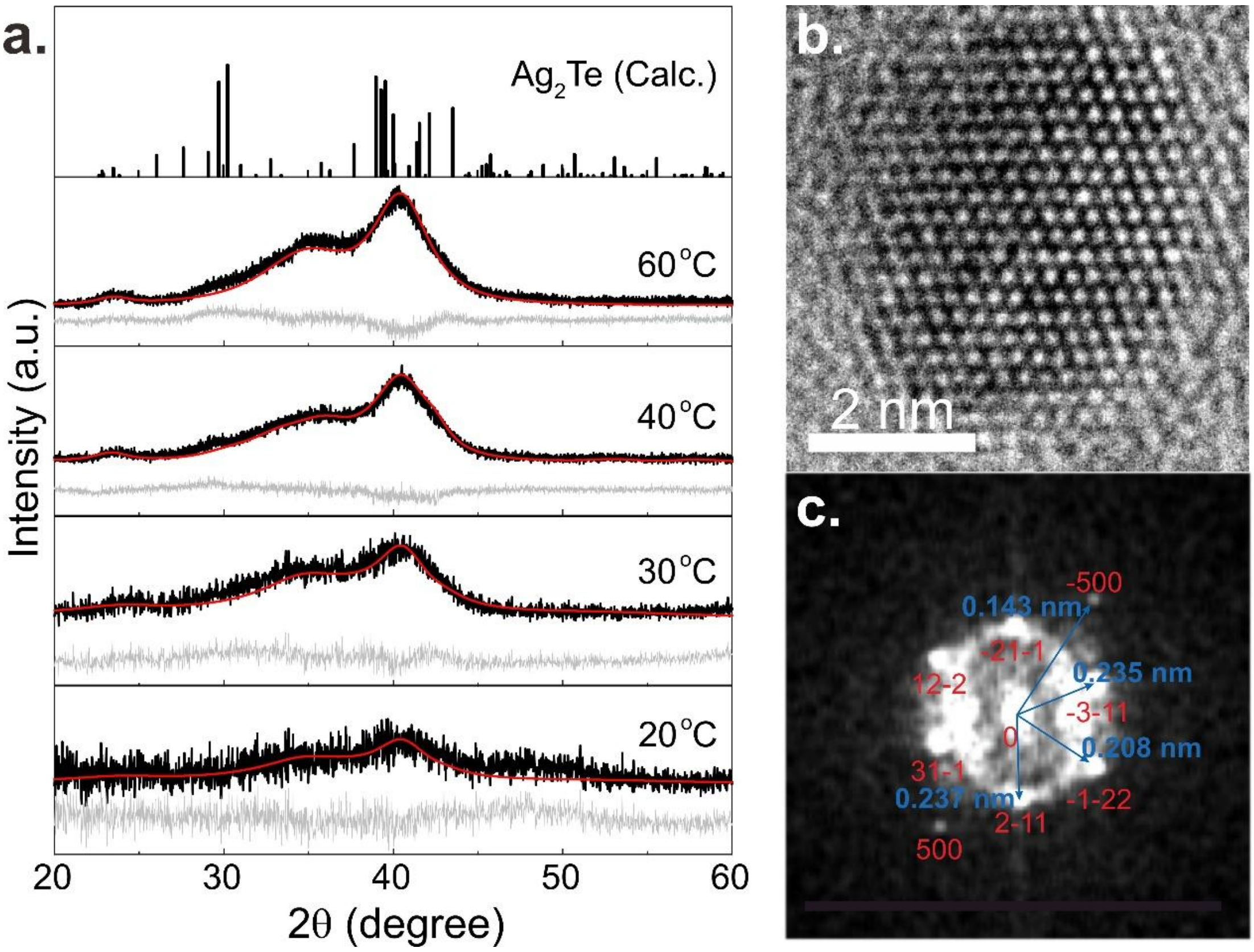

Figure S6. a. Full-profile refinement of the XRD patterns of the Ag_2Te NCs shown in Figure 1h. The black, red and grey solid lines represent the experimental data, the calculated profile and the difference (residual) curve, respectively. The vertical black bars show the reference bulk monoclinic Ag_2Te pattern (calculated). **b.** Magnified HRTEM image shown in the inset in Figure 1e. **c.** Fast Fourier transform of the image in panel b, with the main planes indexed along [011] zone axis.

6. Atomic composition of Ag_2Te NCs

Table S3. Atomic composition in [%] of Ag_2Te NCs synthesised at 60 °C with different Ag:Te ratios, determined from survey XPS spectra.

Ag:Te ratio by load	Batch	Ag	Te	S	O	C	Experimental Ag:Te ratio
4:1 Ag-rich	1	27.9	5.8	10.6	4.4	51.3	**4.8**
	2	39.6	9.9	17.1	5.1	28.3	**4.0**
	3	49.0	12.3	19.1	3.4	15.2	**4.0**
	4	43.9	12.8	17.3	3.8	22.2	**3.4**
2:1 st-Ag_2Te	1	38.0	17.6	23.5	4.8	16.0	**2.2**
	2	24.3	9.7	11.0	8.7	46.2	**2.5**
	3	22.8	9.1	9.8	6.7	51.7	**2.5**
	4	35.4	15.4	12.0	6.6	30.7	**2.3**

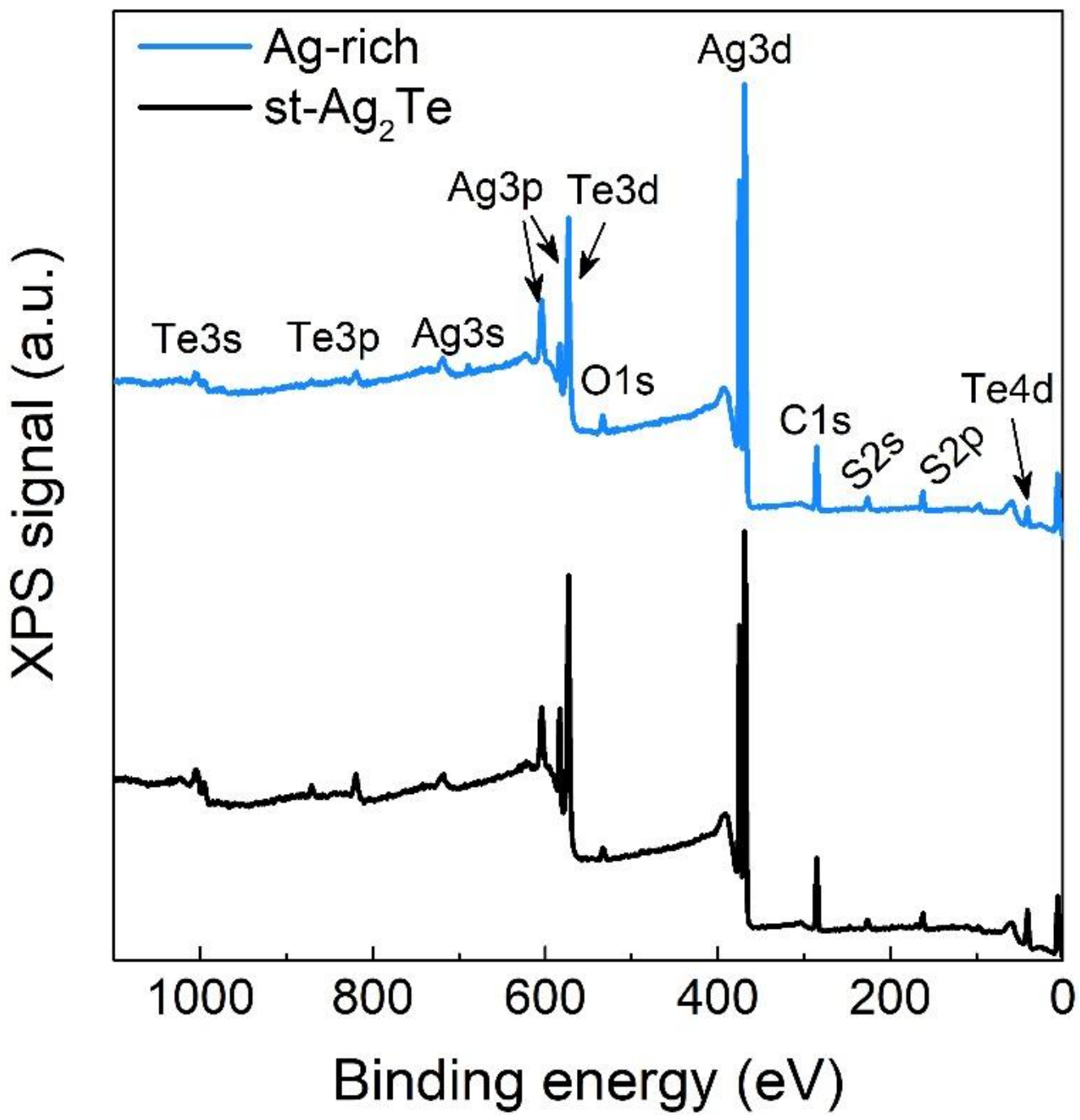

Figure S7. XPS survey spectra of Ag-rich and st-Ag_2Te NCs. The spectrum of st-Ag_2Te is the same as in Figure 2a, and presented for comparison.

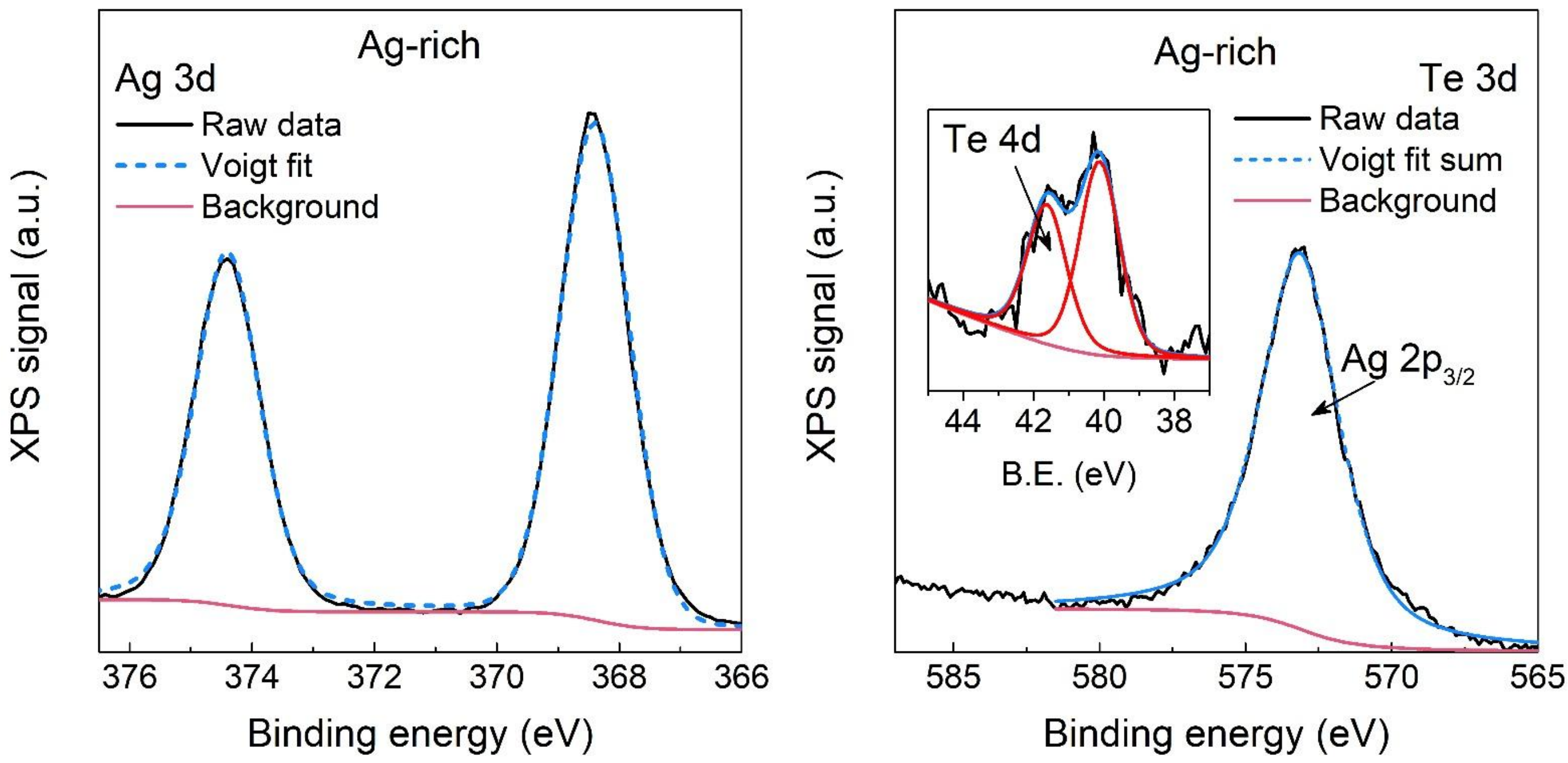

Figure S8. High-resolution XPS spectra of Ag 3d (**a**), and Te 3d (**b**) core levels for the Ag-rich NCs, complementary to stoichiometric composition shown in **Figure 2b and c**. The inset in (**b**) shows the XPS spectrum of Te 4d core levels.

7. Photodetector characterization

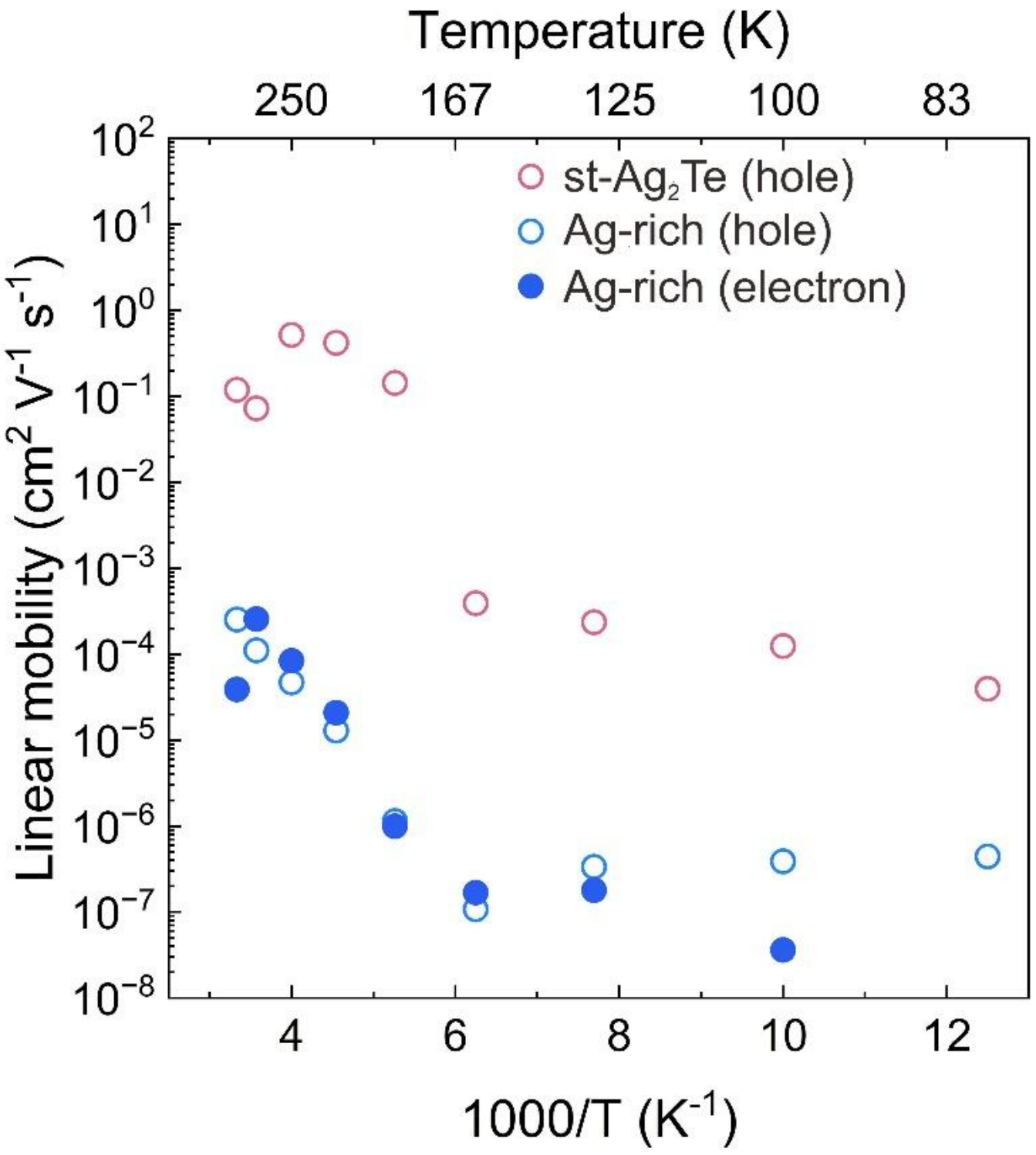

Figure S9. Charge carrier mobility of Ag_2Te NC films with different Ag:Te ratio, as a function of the inverse temperature.

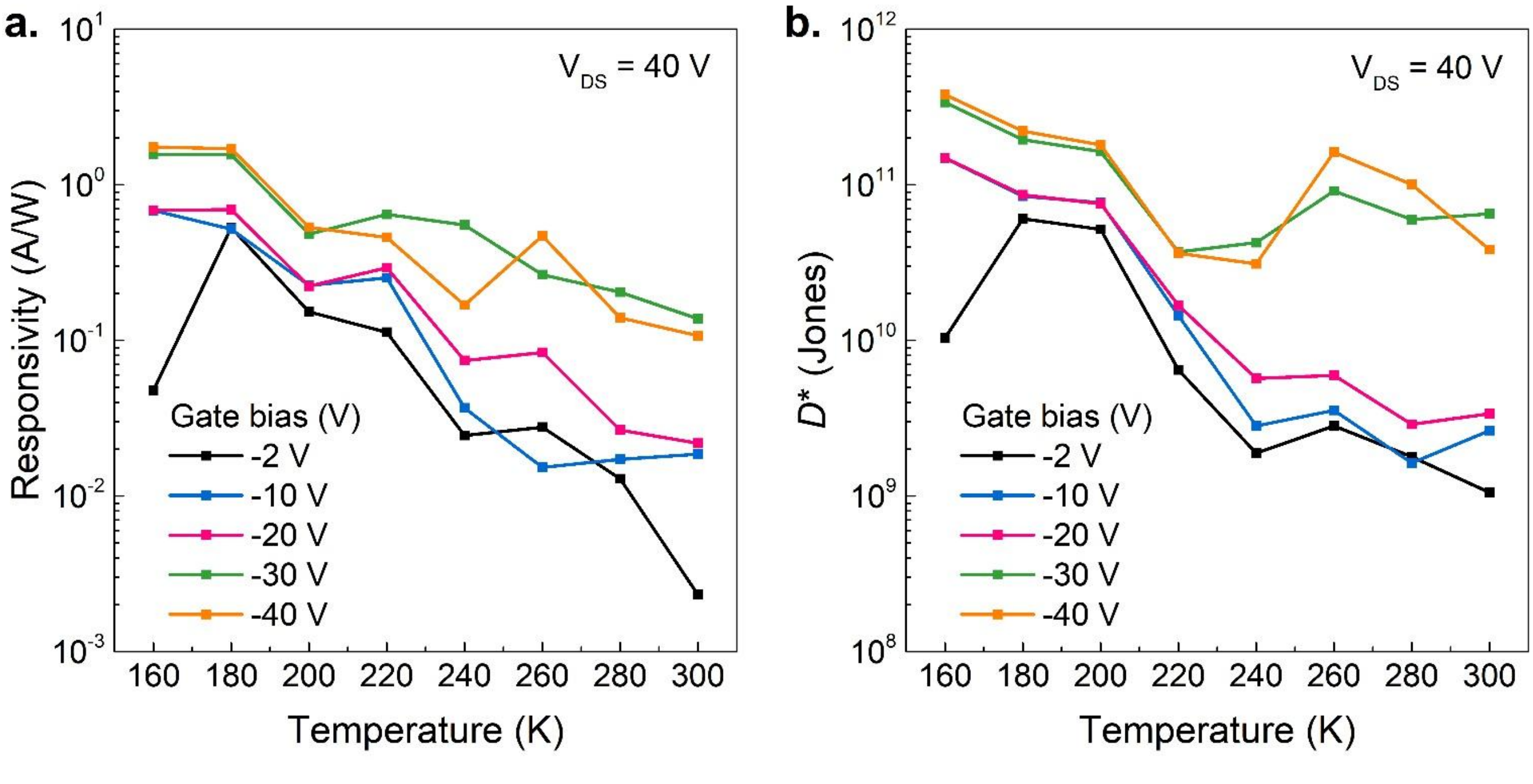

Figure S10. a. Responsivity and **b.** specific detectivity D^* of FET photodetector with n-type Ag_2Te NC film as an active layer under a broadband illumination (calibrated 2796 K blackbody) filtered through 1000 nm long-pass, as a function of the operating temperature and gate bias voltage.

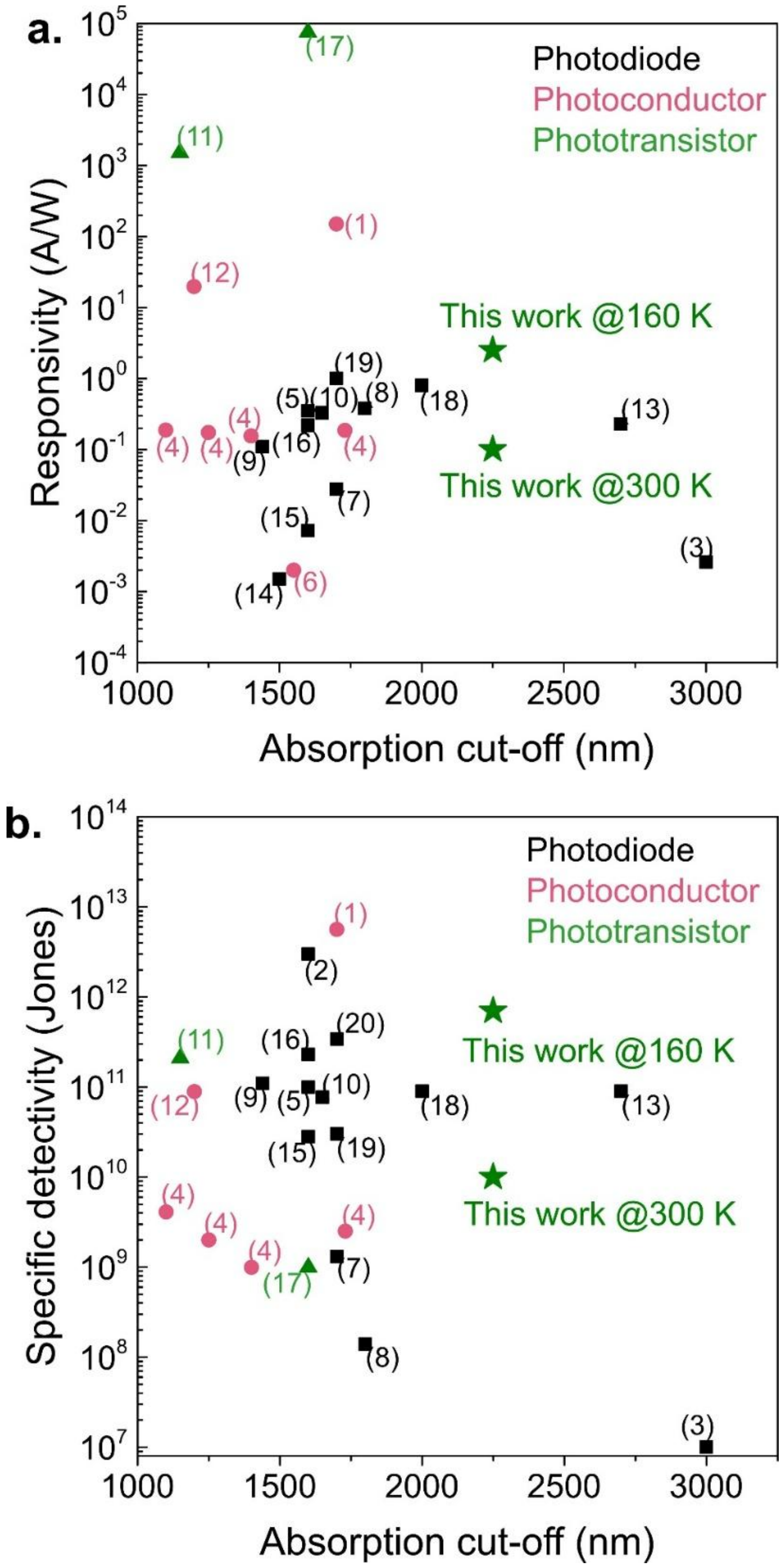

Figure S11. a. Responsivity and **b.** specific detectivity of several Ag_2Te NCs-based devices reported recently, compared with values achieved in this work.

Reference list for the literature provided in Figure S11

(1) Mao, C.; Yao, F.; Aleksandrov, D.; Liu, F.; Wu, W. Silver Telluride Quantum Dots on Silicon Near-Infrared Photodetectors. ACS Appl. Mater. Interfaces 2025, 17, 31230-31236.

(2) Wang, Y.; Peng, L.; Schreier, J.; et al. Silver Telluride Colloidal Quantum Dot Infrared Photodetectors and Image Sensors. Nat. Photonics 2024, 18, 236-242.

(3) Ahn, Y.; Eom, S. Y.; Kim, G.; Lee, J. H.; Kim, B.; Kim, D.; Si, M.-J.; Yang, M.; Jung, Y.; Kim, B. S.; Chung, Y. J.; Jeong, K. S.; Baek, S.-W. Silver Telluride Colloidal Quantum Dot Solid for Fast Extended Shortwave Infrared Photodetector. Adv. Sci. 2024, 11, 2407453.

(4) Park, S. Y.; Paul, S. J.; Li, Z.; et al. Quad-Band Short-Wave Infrared Detection Using Bandgap-Controlled Ag_2Te Quantum Dots. Adv. Funct. Mater. 2026, 36, e12679.

(5) Wang, Y.; Wu, H.; Rodà, C.; Peng, L.; Taghipour, N.; Dosil, M.; Konstantatos, G. Shortwave Infrared Light Detection and Ranging Using Silver Telluride Quantum Dots. Adv. Mater. 2025, 37, 2500977.

(6) Kim, H.-N.; Yoo, D.; Sharma, A.; Choi, M.-J. Continuous monomer supply for highly monodisperse Ag_2Te colloidal quantum dots for SWIR photodetectors. Mater. Horiz. 2025, 12, 6342-6348.

(7) Sharma, A.; Yoo, D.; Kim, H.-N.; Choi, M.-J. Sequential Surface Tailoring from Colloid to Solid in Ag_2Te Colloidal Quantum Dots Enables High Hole Mobility and Efficient Shortwave Infrared Photodetection. Nano Energy 2025, 141, 111091.

(8) Yuan, D.; Cao, F.; Han, Z.; Liu, M.; Deng, X.; Zhang, L.; Liu, X.; Cao, S.; Liu, X.; Yang, Z.; Deng, Z.; Li, Y.; Voznyy, O.; Fan, Q.; Sun, B.; Huang, W. Zinc Halide Enables Highly Monodisperse Ag_2Te Colloidal Quantum Dots for Short-Wave Infrared Photodetectors. Nano Energy 2025, 140, 111026.

(9) Lee, J. A.; Lee, I. S.; Kang, D.; Kim, N.; Kim, J.; Baek, S.-W.; Kim, Y. Chemical Reactivity-Controlled Synthesis of Silver Chalcogenide Colloidal Quantum Dots for Efficient Shortwave Infrared Photodetectors. Small 2025, 21, 2412420.

(10) Dai, P.; Wang, Z.; Tang, Z.; Zhang, Z.; Wang, Y.; Yang, X.; Jiang, J.; Zhang, Y.; Yang, H. Bismuth-Doped Ag_2Te Colloidal Quantum Dots for High-Performance Shortwave-Infrared Photodetectors. Inorg. Chem. 2025, 64, 14637-14643.

(11) Pan, Z.; Li, J.; Dai, W.; Gou, Y.; Yu, H.; Tang, W. A high-performance solution-processed near-infrared phototransistor with a non-toxic Ag_2Te colloidal quantum dots/ZnO heterostructure channel. J. Mater. Chem. C 2025, 13, 12193-12198.

(12) Lai, Y.; Luo, J.; Zhao, X.; Chen, H.; Song, Q.; Zheng, K.; Tong, X.; Wang, R.; Zhou, Y. Plasmon-enhanced colloidal silver telluride quantum dots for boosted near-infrared photodetection performance. J. Mater. Chem. A 2026, 14, 3490-3503.

(13) Kim, G.; Choi, D.; Eom, S. Y.; Jung, E. D.; Lee, J. H.; Rehl, B.; Kim, S. Y.; Hoogland, S.; Sargent, E. H.; Jeong, K. S. Extended Short-Wavelength Infrared Ink by Surface-Tuned Silver Telluride Colloidal Quantum Dots and Their Infrared Photodetection. ACS Mater. Lett. 2024, 6, 4988-4996.

(14) Ouyang, J.; Graddage, N.; Lu, J.; Zhong, Y.; Chu, T.-Y.; Zhang, Y.; Wu, X.; Kodra, O.; Li, Z.; Tao, Y.; Ding, Ag_2Te Colloidal Quantum Dots for Near-Infrared-II Photodetectors. J. ACS Appl. Nano Mater. 2021, 4, 13587-13601.

(15) Tang, X.; Jiang, Y.; Duan, Z.; Bu, Y.; Zeng, W.; Yang, J.; Yuan, Z.; Tai, H. Surface ligand engineering of Ag_2Te colloidal quantum dots for a self-powered shortwave infrared photodetector. J. Mater. Chem. C 2025, 13, 21418-21427.

(16) Wang, Z.; Tang, Z.; Zhang, Z.; Zhang, Y.; Jiang, J.; Yang, H.; Wang, Q. Interface modification for silver telluride quantum dots shortwave-infrared photodetectors beyond 1.5 μm. Nano Research, 2026, 19 (8): 94908647.

(17) Jeong, S.-J.; Ko, H. W.; Jo, S.; Selvaraj, J.; Kim, J.-M.; Lee, N.; Ahn, J.-H.; Lee, H.; Jeong, S.; Kim, H.; Seo, P. H.; Shim, J.W.; Kim, Y.; Ra, H.-S.; Park, M.-C.; Lee, J.-S. High-Gain Ag_2Te/MoS_2 Hybrid Photodetectors for Short-Wave Infrared Imaging. Adv. Mater. 2026, 38, e20984.

(18) Rastogi, P.; Izquierdo, E.; Gréboval, C.; Cavallo, M.; Chu, A.; Dang, T. H.; Khalili, A.; Abadie, C.; Alchaar, R.; Pierini, S.; Cruguel, H.; Witkowski, N.; Utterback, J. K.; Brule, T.; Xu, X. Z.; Hollander, P.; Ouerghi, A.; Gallas, B.; Silly, M. G.; Lhuillier, E. J. Extended Short-Wave Photodiode Based on $CdSe/HgTe/Ag_2Te$ Stack with High Internal Efficiency. Phys. Chem. C 2022, 126, 13720-13728.

(19) Zhao, P.; Mu, G.; Wen, C.; Qi, Y.; Lv, H.; Tang, X. Colloidal Quantum Dots-Based Three-Band Infrared Photodetector with the Bias-Tunable Spectral Response. Phys. Status Solidi RRL 2024, 18, 2300121.

(20) Wang, B.; Yuan, M.; Liu, J.; Zhang, X.; Liu, J.; Yang, J.; Gao, L.; Zhang, J.; Tang, J.; Lan, X. Synergism in Binary Nanocrystals Enables Top-Illuminated HgTe Colloidal Quantum Dot Short-Wave Infrared Imager. Nano Lett. 2024, 24, 9583-9590.
